# The role of a low protein diet supplemented with ketoanalogues on kidney progression in pre-dialysis chronic kidney disease patients

**DOI:** 10.1038/s41598-023-42706-w

**Published:** 2023-09-19

**Authors:** Saravanee Ariyanopparut, Kamonchanok Metta, Yingyos Avihingsanon, Somchai Eiam-Ong, Piyawan Kittiskulnam

**Affiliations:** 1 Department of Medicine, Faculty of Medicine, Chulalongkorn University, Bangkok, Thailand; 2 Division of Nephrology, Department of Medicine, Faculty of Medicine, Chulalongkorn University, Bangkok, Thailand; 3 Division of Internal Medicine-Nephrology, Department of Medicine, Faculty of Medicine, Chulalongkorn University and King Chulalongkorn Memorial Hospital, Thai Red Cross Society, Bangkok, 10330 Thailand

**Keywords:** Medical research, Nephrology

## Abstract

In slowing kidney progression, numerous pre-dialysis chronic kidney disease (CKD) patients could not adhere to the well-established dietary pattern, including a very low protein diet, 0.3–0.4 g/kg/day, plus a full dose ketoanalogues (KAs) of amino acids. We evaluated the role of a low protein diet (LPD), 0.6–0.8 g/kg/day, combined with KAs (LPD–KAs) on CKD progression. We extracted data in the retrospective cohort using electronic medical records (n = 38,005). Participants with LPD–KAs for longer than six months were identified. An unmatched control group, LPD alone, was retrieved from the same database. Cox proportional hazard models were performed to examine the associations between LPD–KAs and outcomes. The primary outcome was either a rapid estimated glomerular filtration rate (eGFR) decline > 5 mL/min/1.73m^2^/year or commencing dialysis. Other secondary outcomes include changes in proteinuria, serum albumin, and other metabolic profiles were also assessed. A total of 1042 patients were finally recruited (LPD–KAs = 543). Although patients with LPD–KAs had significantly lower eGFR and a prevalence of diabetes, age, and dietary protein intake were comparable between LPD–KAs (0.7 ± 0.2 g/kg/day) and LPD alone groups (0.7 ± 0.3 g/kg/day, *p* = 0.49). During a median follow-up of 32.9 months, patients treated with LPD–KAs had a significantly lower risk of kidney function decline (HR 0.13; 95% CI 0.09–0.19, *p* < 0.001) and dialysis initiation (HR 0.24; 95% CI 0.12–0.49, *p* < 0.001) than LPD alone after adjusting for confounders. The annual rate of eGFR decline in patients receiving LPD–KAs was 4.5 [3.4–5.5] mL/min/1.73m^2^ compared with 7.7 [6.0–9.4] mL/min/1.73m^2^ in LPD alone (*p* = 0.001). According to KAs dose–response analysis, the daily dose of ≤ 5 tablets was conversely associated with a higher risk of the primary endpoint, whereas the association disappeared among patients receiving a dose of > 6 tablets. The spot urine protein creatinine ratio and serum phosphate levels were not significantly different between groups. LPD–KAs could retard kidney progression compared with LPD alone. This favorable effect was significant among CKD patients receiving a daily KAs dose of more than six tablets. Future randomized controlled trials should be performed to verify these findings.

## Introduction

Chronic kidney disease (CKD), defined as an irreversible abnormality of kidney function, has a high estimated prevalence of between 11 and 13% worldwide, with the vast majority having an estimated glomerular filtration rate (eGFR) of less than 60 mL/min/1.73 m^2^^1^. Dialysis remains the treatment option for most individuals with kidney failure as CKD becomes advanced but is associated with a high economic burden, markedly reduced quality of life, and decreased overall survival^2^. Therefore, the main task in nephrology practice is to develop several strategies to avoid or defer dialysis. In addition to pharmacologic intervention, nutritional management with restricted dietary protein intake plays an important role in slowing CKD progression^3^. The findings from an experimental model demonstrated that a high-protein diet was significantly associated with a glomerulomegaly-induced higher degree of albuminuria compared with a low protein isocaloric diet^4^. Previous studies in CKD patients also indicated the beneficial effects of dietary protein restriction on the retardation of kidney function decline^5–7^. Moreover, a recent meta-analysis among both diabetic and non-diabetic CKD patients revealed that a low protein diet (LPD) of less than 0.6–0.8 g/kg/day was significantly associated with a lower incidence rate of end-stage kidney disease (ESKD) requiring dialysis^8^.

According to the updated Kidney Disease Outcomes Quality Initiative (KDOQI) nutrition guideline^9^, a very low protein diet (VLPD) providing 0.3–0.4 g dietary protein/kg/day supplemented with ketoanalogues (KAs) of essential amino acids may be considered as another dietary regimen to reduce the risk of ESKD. Due to the lack of an amino group in the chemical structure, KAs can be utilized in place of their respective amino acids without releasing nitrogen waste products into the body^10^. With a VLPD approach, the full dose of additional KAs containing 0.125 g of keto acids/kg/day (approximately one tablet per 5 kg per day) has been suggested to meet the minimal protein requirement in pre-dialysis CKD patients^11^. Previous randomized controlled studies among non-diabetic CKD populations^11–13^ have reported that the rate of CKD progression and dialysis initiation in patients receiving VLPD supplemented with KAs, corresponding to up to 12 tablets/day, was significantly lower than in the LPD group alone. However, adherence to a VLPD regimen in real-world implementation seems to be relatively low^14^. Although the nitrogen balance and adaptive protein metabolism are maintained despite supplementing VLPD with KA, protein-energy wasting may occur in the setting of inadequate daily energy intake, particularly without close nutritional monitoring^15^.

Unlike its mandatory use in VLPD, the supplementation of KAs in LPD is optional as recommended by the authorities’ consensus^16^. An earlier 6-month observational study found that an LPD supplemented with KAs (LPD–KAs) regimen was associated with a less steep kidney function decline than LPD alone and could enhance patients’ compliance with the diet protocol^17^. However, scarce data are available regarding the extended period of LPD–KAs use among pre-dialysis CKD. Furthermore, the appropriate dose of KAs administered along with LPD alone is yet to be determined. In the present study, we conducted a cohort of pre-dialysis CKD patients and analyzed data, including the assessment of serum creatinine, eGFR, and dietary protein intake, as well as other significant metabolic profiles. The main goals of this study were to compare the rate of kidney function decline and dialysis initiation between CKD patients receiving LPD–KAs and LPD alone and to evaluate the optimal dose of KAs used for supplementation in the LPD–KAs dietary regimen.

## Materials and methods

### Study design and participants

**Figure 1:**
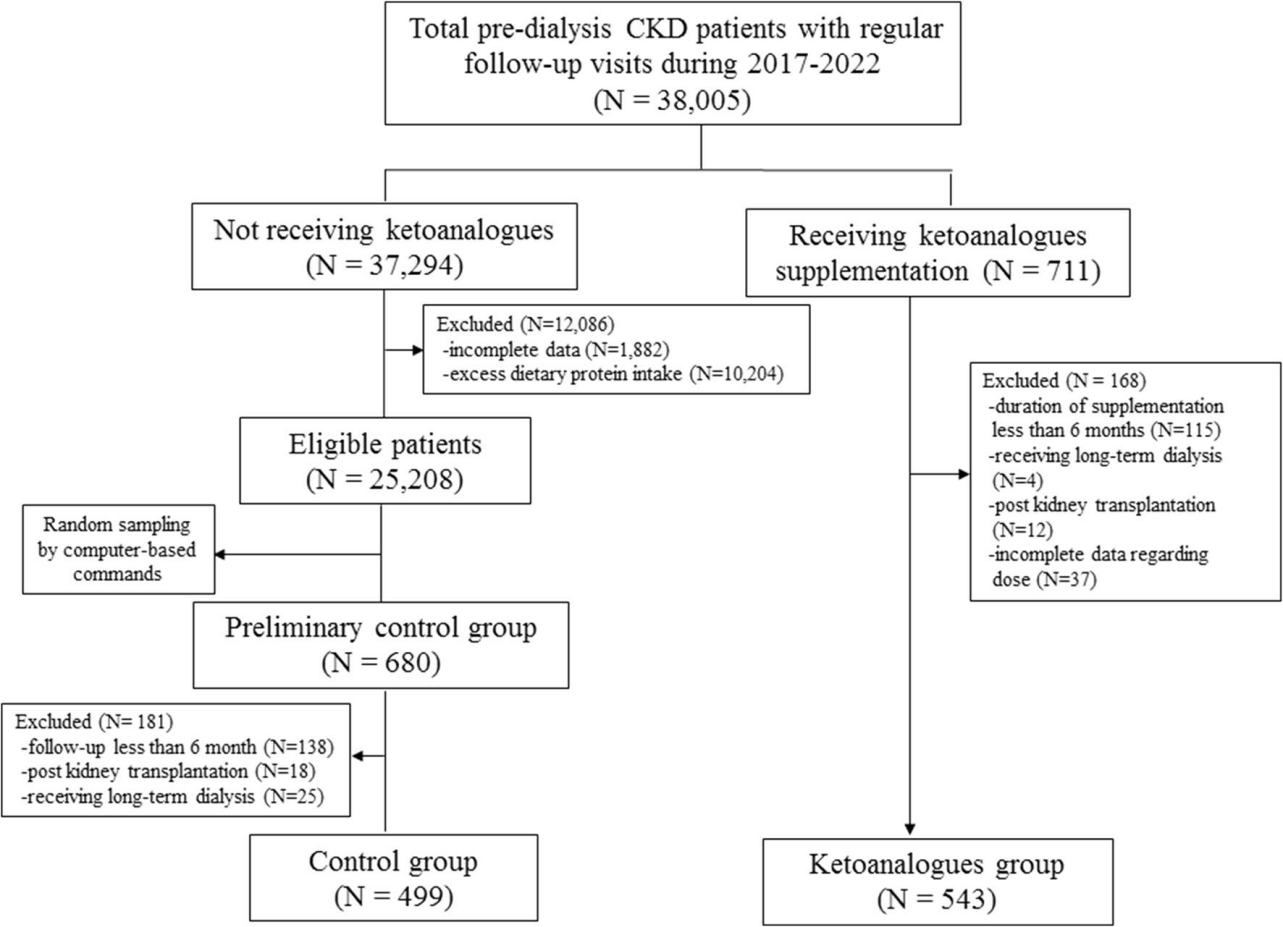
Flow chart of the study.

This was a retrospective cohort study of pre-dialysis CKD patients at our tertiary care facility (King Chulalongkorn Memorial Hospital, Bangkok, Thailand). We extracted all longitudinal data from both the general medicine and nephrology outpatient clinics’ database using electronic medical records via the High-Technology Information System (HIS). The HIS is an online operating system comprising clinic visits, hospitalizations, medical procedures, and medication uses. Eligible participants were over 18 years old, received regular follow-up visits during the past 12 months, had eGFR calculated by the Chronic Kidney Disease Epidemiology Collaboration (CKD-EPI) equation^18^, and were not yet on long-term kidney replacement therapy (KRT). The exclusion criteria included participants with post-kidney transplantation, a history of acute kidney injury in the preceding three months, over 90 years of age, pregnant, and breastfeeding. Based on the Comptroller General’s Department, Ministry of Finance of Thailand, KAs could be reimbursed for prescription drug coverage in the case of government and state enterprise officers having a CKD diagnosis and achieving a restricted protein diet of less than 40 g/day. The dosing prescription of KAs was independently adjudicated by the patients’ attending physicians. Pre-dialysis CKD patients receiving LPD–KAs (Ketosteril®) for longer than six months were identified utilizing the online medical reconciliation system. To alleviate the imbalanced confounders between groups that might have potential impact on eGFR deterioration such as the stage of CKD or dietary protein intake in this study, an unmatched control group without KAs supplementation, LPD alone, was retrieved by random sampling from the same database. We initially excluded CKD patients with excess dietary protein intake followed by stratification of patients into groups according to their CKD stage. Subsequently, a mathematic coding formula was used to generate random numbers (Fig. 1). This study was approved by the Institutional Review Board of the Faculty of Medicine, Chulalongkorn University (Certificate of approval No. 751/2020) in compliance with the ethical standards of the International guidelines for human research protection as Declaration of Helsinki, the Belmont Report, and International Conference on Harmonization in Good Clinical Practice (ICH-GCP). The present study does not contain any data obtained directly from human (only document extraction) and there were no additional clinical and laboratory studies in the included participants. The need for informed consent has been waived by the Institutional Review Board of the Faculty of Medicine, Chulalongkorn University (IRB No. 284/63).

### Data collection, laboratory assessment, and nutritional education

Data were retrieved from the patients’ medical records, including demographic characteristics, laboratory findings, comorbidities by the 10th revised version of the International Classification of Disease (ICD-10) code, and current medications. Body weight was recorded to the nearest 0.1 kg for each visit, with height measured using a stadiometer. Systolic and diastolic blood pressures were recorded by standardized office blood pressure measurements. We calculated the body mass index (BMI) using body weight divided by the square of height in meters. The serum creatinine level and eGFR were determined as markers of kidney function. Serum creatinine was measured using the Roche Diagnostics (Indianapolis, IN) enzymatic assay on a COBAS INTEGRA® 400 plus analyzer. GFR was estimated after enzymatic creatinine values were calibrated by traceable high-level isotope dilution mass spectroscopy (IDMS) reference serum creatinine. CKD was defined as any functional or structural abnormalities in kidney function, and stages 1, 2, 3, 4, and 5 were classified based on eGFR levels as follows: ≥ 90, 60–89, 30–59, 15–29, and < 15 mL/min/1.73 m^2^ and not receiving KRT, respectively. Untimed urinary albumin-to-creatinine ratios (UACR) of 30–299 mg/g and > 300 mg/g were considered as having albuminuria category A2 and A3, respectively^19^. We also collected other metabolic laboratories, including electrolytes, lipid profiles, fasting plasma glucose, and hemoglobin A1C levels.

For nutritional education, all pre-dialysis CKD patients were routinely instructed with standardized general knowledge regarding a CKD diet. In order to receive an individualized meal plan, specific counseling by a well-trained dietitian was also provided upon the patient’s request without a copayment. Both diabetic and non-diabetic CKD patients were informed to comply with protein restriction between 0.6 and 0.8 g/kg/day while maintaining an adequate daily energy intake of 25–35 kcal/kg/day. For patients with lower and upper BMI extremes, ideal body weight was used instead of actual edema-free body weight to calculate the nutritional targets. Nutritional compliance to the protein diet was regularly checked either by dietary recall or dietary protein intake estimated by the calculation of normalized protein equivalent of total nitrogen appearance (nPNA) in 24-h urine collection using the Maroni formula^20^.

### Kidney function follow-up and outcome measures

We evaluated the association of LPD–KAs with the absolute changes in kidney function over time with the index date of study on January 1, 2017. Rapid CKD progression was defined as an annual decline in eGFR of more than 5 mL/min/1.73 m^2^, according to the Kidney Disease Improving Global Outcomes (KDIGO)^19^. The last dose of KAs during the preceding 90 days before the outcomes or end of the study was used for analysis. In order to measure the observational period until the occurrence of study outcomes (failure) or the end of study (censor) with different index dates among each participant, all analysis were performed in terms of time becoming at risk (analysis time) using survival-time command. We censored the follow-up time at the start of long-term dialysis, kidney transplantation, or the end of the study (March 31, 2022), whichever occurred first. The primary endpoint was the composite of either rapid CKD progression or commencement of dialysis. Among KAs users, we also sought to find the optimal KAs dosage that associated with the reduction of primary endpoint. The secondary endpoints were changes in serum biochemistry, including albumin and electrolyte, as well as proteinuria levels.

### Statistical analysis

We presented the patient characteristics using mean ± standard deviation (SD) for normally distributed or median (25th–75th percentile), non-normally distributed variables, and proportions of discrete variables. We compared the patients’ characteristics using chi-squared, unpaired t-tests, and Mann–Whitney U test as appropriate. Categorical data were described in proportions and percentages. Differences between variables were compared using unpaired Student *t-*tests for normally distributed variables or Mann–Whitney *U* tests (Wilcoxon rank–sum test) for non-normally distributed variables. We used the Cox proportional hazards model to estimate the relationship between the use of KAs and primary outcomes. Selected variables were analyzed by both univariable and multivariable models (data presented as [hazard ratio (HR); 95% confidence interval (CI)]). We examined a model that included age and sex (model 1). We then added diabetes mellitus, coronary artery disease, cerebrovascular disease, hypertension as comorbidities, and the use of RAS blockers (model 2). We further incorporated serum albumin, bicarbonate, and HbA1C levels as a final model (model 3). We conducted all analyses in Stata 15 (StataCorp LP, College Station, TX), and *P* values of less than 0.05 were considered statistically significant.

## Results

### Baseline demographic data of participants

A total of 38,005 patients with diabetic and non-diabetic pre-dialysis CKD were extracted from general medicine (67.9%) and nephrology (32.1%) clinics. Of these, 1042 CKD patients were finally recruited, with 543 patients being treated with LPD–KAs (Fig. 1). All enrolled participants were still alive during the entire study period. The characteristics of the participants are shown in Table 1. The mean age was 72.1 ± 13.2 years and was comparable between patients with LPD–KAs and LPD alone. Approximately half of the patients had diabetes (44.4%), and 46.6% were women. The average body mass index was 24.9 ± 4.7 kg/m^2^, and 54.9% of patients had albuminuria category A3. Twenty-seven percent of patients received renin-angiotensin system-blocking agents. The median eGFR was 31.3 (18.5–47.1) mL/min/1.73 m^2^, and 87.5% of participants had an eGFR of less than 60 mL/min/1.73 m^2^. There were no significant differences in the proportions of participants classified into CKD stage 3 (*p* = 0.42) and 4 (*p* = 0.08) between patients receiving LPD–KAs and LPD alone. Overall, the participants in the whole study complied with the LPD regimen, and the estimated dietary protein intake was comparable between patients with LPD–KAs (0.7 ± 0.2 g/kg/day) and LPD alone (0.7 ± 0.3 g/kg/day) groups at baseline of the study (*p* = 0.49).

**Table 1:** Patient characteristics at baseline of the study.

Parameters	Total (n = 1042)	LPD (n = 499)	LPD–KAs (n = 543)	*P* value
Age, years	72.1 ± 13.2	71.6 ± 13.6	72.5 ± 12.9	0.22
Men, n (%)	557 (53.4)	249 (49.9)	308 (56.7)	0.03
Diabetes mellitus, n (%)	436 (44.4)	266 (53.3)	197 (36.3)	< 0.001
Hypertension, n (%)	418 (40.1)	250 (50.1)	168 (30.9)	< 0.001
Cardiovascular disease, n (%)	123 (11.8)	62 (12.4)	61 (11.2)	0.55
Preexisting cancer, n (%)	113 (10.8)	76 (15.2)	37 (6.8)	< 0.001
Receiving ACEI or ARB, %	282 (27.1)	159 (31.9)	123 (22.7)	0.001
Systolic blood pressure, mmHg	136.0 ± 20.5	135.9 ± 20.6	136.2 ± 20.4	0.83
Diastolic blood pressure, mmHg	71.7 ± 12.2	71.8 ± 12.5	71.6 ± 12.1	0.84
Body weight, kg	63.8 ± 14.2	62.5 ± 13.9	64.9 ± 14.3	0.01
Body mass index*, kg/m^2^	24.9 ± 4.7	24.6 ± 4.7	25.3 ± 4.6	0.08
Blood urea nitrogen, mg/dL	34.1 ± 21.6	32.7 ± 23.3	35.4 ± 19.8	0.04
Serum creatinine, mg/dL	3.2 ± 4.9	2.7 ± 3.1	3.5 ± 6.1	0.003
eGFR, mL/min/1.73 m^2^	31.3 (18.5–47.1)	35.9 (22.3–53.4)	28.9 (15.9–41.1)	< 0.001
CKD stage 3, n (%)	419 (40.2)	204 (41.5)	212 (39.0)	0.42
CKD stage 4, n (%)	292 (28.0)	127 (25.4)	165 (30.4)	0.08
CKD stage 5, n (%)	201 (19.3)	77 (15.4)	124 (22.8)	0.002
Serum sodium, mmol/L	137.9 ± 4.1	137.7 ± 4.6	138.2 ± 3.6	0.16
Serum potassium, mmol/L	4.3 ± 0.6	4.2 ± 0.6	4.3 ± 0.6	0.06
Serum bicarbonate, mmol/L	22.9 ± 3.5	22.6 ± 3.6	22.9 ± 3.4	0.38
Serum calcium, mg/dL	9.1 ± 0.7	9.1 ± 0.1	9.0 ± 0.7	0.90
Serum phosphate, mg/dL	3.7 ± 1.2	3.8 ± 1.5	3.6 ± 0.9	0.01
Serum magnesium, mmol/L	0.9 ± 0.2	0.8 ± 0.1	0.9 ± 0.2	0.04
Serum albumin, g/L	3.8 ± 0.5	3.7 ± 0.6	3.8 ± 0.4	0.002
Serum total cholesterol, mg/dL	176.6 ± 49.2	180.2 ± 51.7	173.8 ± 47.1	0.05
Serum triglyceride, mg/dL	135.5 ± 78.2	136.3 ± 76.6	134.8 ± 79.4	0.75
Serum low-density lipoprotein, mg/dL	98.1 ± 37.0	102.9 ± 39.6	94.3 ± 34.5	0.001
Fasting blood sugar, mg/dL	120.6 ± 47.3	121.1 ± 43.5	120.3 ± 50.2	0.82
Hemoglobin A1C, %	6.2 ± 1.4	6.3 ± 1.5	6.1 ± 1.3	0.01
Protein intake^†^, g/kg/day	0.7 ± 0.2	0.7 ± 0.3	0.7 ± 0.2	0.49
UACR, mg/g creatinine	492.6 (48.6–1740.3)	338.3 (39.3–1443.9)	609.7 (88.1–2003.3)	0.03

ACEI, angiotensin converting enzyme inhibitor; ARB, angiotensin receptor blockers; eGFR, glomerular filtration rate estimated by Chronic Kidney Disease Epidemiology Collaboration (CKD-EPI) equation; LPD, low protein diet; KAs, ketoanalogues of amino acids; UACR, spot urinary albumin creatinine ratio.

Data are presented as mean (SD) and median (25th–75th).

*P* < 0.05 consider significantly different between LPD and LPD–KAs groups.

*Calculated by body weight (in kg) divided by the square of height (in meters).

^†^Calculated by normalized protein equivalent of total nitrogen appearance in 24-h urine collection.

### Association of KAs usage and the composite primary endpoints

**Figure 2:**
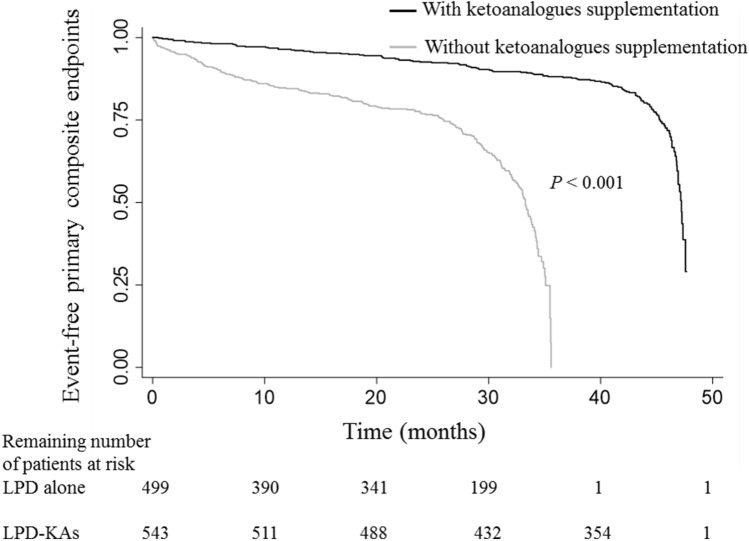
Kaplan–Meier estimates of primary composite endpoint, defined as an annual eGFR decline of more than 5 mL/min/1.73 m^2^ or long-term dialysis initiation, according to CKD patients receiving low-protein diet with and without ketoanalogues supplementation. *P* value using the log-rank test indicates between groups comparison.

During the median follow-up period of 32.9 months, 333 primary outcome events including the composite of rapid eGFR decline or long-term dialysis initiation were observed (32.1%). The median follow-up duration in the LPD and LPD–KAs was 28.2 and 43.3 months, respectively. The primary outcome incidence rate was 10.4 per 1000 person-months (95% CI 9.4–11.6). Patients with LPD–KAs had a significantly lower primary outcome (6.1 per 1000 person-months; 95% CI 5.1–7.2) compared to those with LPD alone (18.3 per 1000 person-months; 95% CI 15.9–20.9) (Fig. 2). Patients receiving LPD–KAs had a significantly lower risk of the primary outcome (unadjusted HR 0.12; 95% CI 0.09–0.17, *p* < 0.001), and this association persisted after adjusting for potential confounders including age, sex, diabetes status, comorbidities, and laboratory parameters (Table 2). Furthermore, the significant association between LPD–KAs and the primary outcome was not modified by diabetes status (Table 3). For the analysis of an individual primary outcome component, the likelihood of developing rapid eGFR decline was significantly lower among CKD patients with LPD–KAs when compared with the LPD alone group (adjusted HR 0.13; 95% CI 0.09–0.19, *p* < 0.001). The rate of annual eGFR decline was significantly slower in patients receiving LPD–KAs (4.5 [3.4–5.5] mL/min/1.73 m^2^) compared to those with LPD alone (7.7 [6.0–9.4] mL/min/1.73 m^2^, *p* = 0.001). In the final adjusted analysis, the rate of long-term dialysis initiation was also significantly lower among CKD patients with LPD–KAs relative to those with LPD alone (adjusted HR 0.24; 95% CI 0.12–0.49, *p* < 0.001) (Table 2).

**Table 2:** Association analysis of ketoanalogues supplementation and primary outcomes among pre-dialysis CKD patients undergoing low-protein diet.

Methods	Unadjusted		Model 1		Model 2		Model 3	
	HR (95% CI)	*p*	HR (95% CI)	*p*	HR (95% CI)	*p*	HR (95% CI)	*p*
Composite of primary outcome (n = 1042)								
Rapid eGFR decline* OR long-term dialysis initiation	0.12 (0.09–0.17)	< 0.001	0.13 (0.09–0.17)	< 0.001	0.11 (0.08–0.16)	< 0.001	0.14 (0.10–0.20)	< 0.001
Individual component of primary outcome (n = 1042)								
Rapid eGFR decline*	0.11 (0.08–0.16)	< 0.001	0.11 (0.07–0.16)	< 0.001	0.10 (0.07–0.15)	< 0.001	0.13 (0.09–0.19)	< 0.001
Long-term dialysis initiation	0.20 (0.11–0.38)	< 0.001	0.21 (0.11–0.40)	< 0.001	0.18 (0.09–0.34)	< 0.001	0.24 (0.12–0.49)	< 0.001

eGFR, estimated glomerular filtration rate.

HR, hazard ratio; 95% CI, 95% confidence interval.

Model 1: Adjusted for age and sex.

Model 2: Additionally adjusted for comorbidities (including diabetes mellitus, coronary artery disease, cerebrovascular disease, hypertension) and use of angiotensin converting enzyme inhibitor or angiotensin receptor blockers.

Model 3: Further adjusted for laboratory parameters including serum albumin, bicarbonate, and hemoglobin A1C level.

*Defined as eGFR drop more than 5 mL/min/1.73 m^2^ of body surface area according to Kidney Disease Improving Global Outcomes (KDIGO) clinical practice guideline recommendation for chronic kidney disease management.

**Table 3:** Subgroup analysis of associations of ketoanalogues supplementation and primary outcomes among pre-dialysis CKD patients undergoing low-protein diet.

Methods	Unadjusted		Model 1		Model 2		Model 3	
	HR (95% CI)	*p*	HR (95% CI)	*p*	HR (95% CI)	*p*	HR (95% CI)	*p*
Diabetes mellitus (n = 463)								
Rapid eGFR decline* OR long-term dialysis initiation	0.04 (0.02–0.09)	< 0.001	0.05 (0.02–0.09)	< 0.001	0.05 (0.02–0.09)	< 0.001	0.04 (0.02–0.10)	< 0.001
Rapid eGFR decline	0.03 (0.02–0.08)	< 0.001	0.04 (0.02–0.08)	< 0.001	0.04 (0.02–0.09)	< 0.001	0.04 (0.02–0.10)	< 0.001
Long-term dialysis initiation	0.10 (0.03–0.47)	0.003	0.11 (0.03–0.48)	0.003	0.09 (0.02–0.39)	0.001	0.06 (0.01–0.35)	0.002
Without diabetes mellitus (n = 579)								
Rapid eGFR decline* OR long-term dialysis initiation	0.16 (0.10–0.22)	< 0.001	0.16 (0.11–0.23)	< 0.001	0.16 (0.11–0.23)	< 0.001	0.24 (0.15–0.36)	< 0.001
Rapid eGFR decline	0.15 (0.10–0.22)	< 0.001	0.16 (0.10–0.23)	< 0.001	0.15 (0.10–0.23)	< 0.001	0.22 (0.14–0.36)	< 0.001
Long-term dialysis initiation	0.19 (0.09–0.38)	< 0.001	0.20 (0.10–0.41)	< 0.001	0.19 (0.10–0.40)	< 0.001	0.32 (0.14–0.73)	0.007

eGFR, estimated glomerular filtration rate.

HR, hazard ratio; 95% CI, 95% confidence interval.

Model 1: Adjusted for age and sex.

Model 2: Additionally adjusted for comorbidities (including diabetes mellitus, coronary artery disease, cerebrovascular disease, hypertension) and use of angiotensin converting enzyme inhibitor or angiotensin receptor blockers.

Model 3: Further adjusted for laboratory parameters including serum albumin, bicarbonate, and hemoglobin A1C level.

*Defined as eGFR drop more than 5 mL/min/1.73 m^2^ of body surface area according to Kidney Disease Improving Global Outcomes (KDIGO) clinical practice guideline recommendation for chronic kidney disease management.

### Dose of KAs supplementation among LPD–KAs

**Figure 3:**
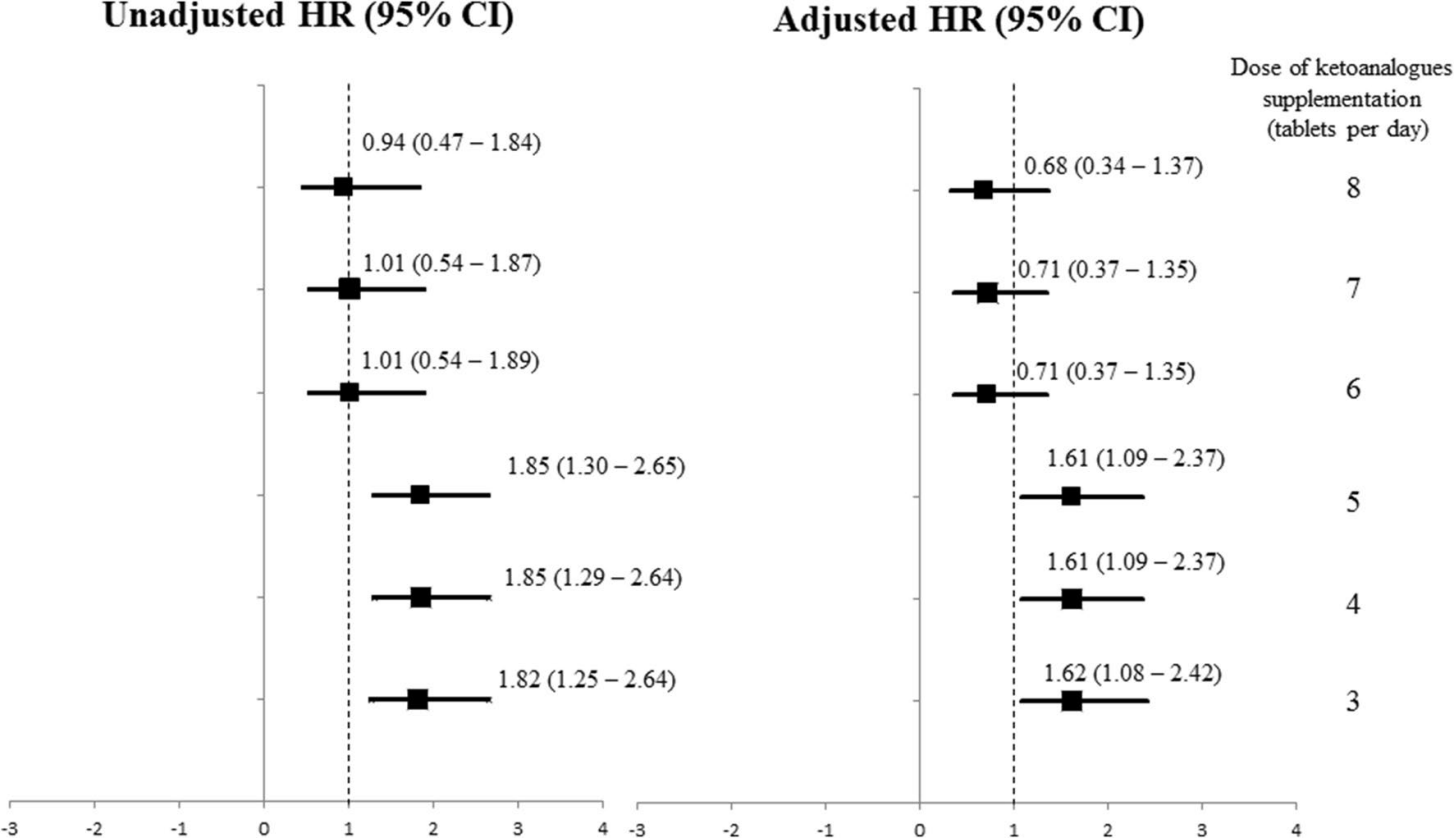
Unadjusted and fully adjusted hazard ration (HR) with 95% confident interval (CI) of primary composite endpoint, defined as an annual eGFR decline of more than 5 mL/min/1.73 m^2^ or long-term dialysis initiation, per an integral increment of daily dose of ketoanalogues among CKD participants receiving supplementation.

The average dose of KAs supplement throughout the study was 4.4 ± 2.1 tablets per day (ranging from 2 to 12 tablets per day). At the end of the study period, the amount of dietary protein intake was still not significantly different between LPD–KAs and the LPD alone group (0.7 ± 0.2 vs. 0.7 ± 0.3 g/kg/day, *p* = 0.97, respectively). Among patients with LPD–KAs, the daily dose of equal or less than five tablets was conversely associated with a higher risk of rapid kidney function decline or commencing dialysis (HR 1.85; 95% CI 1.29–2.64, *p* = 0.001), whereas these associations disappeared among patients receiving a KAs dose of more than six tablets per day (HR 1.01; 95% CI 0.54–1.88, *p* = 0.97) (Fig. 3). The association between the use of KAs at a dose of five tablets per day or less and the primary outcome persisted even after adjusting for confounders (HR 1.61; 95% CI 1.09–2.37, *p* = 0.02). In the final model, the association between a daily dose of KAs of more than seven and eight tablets and the primary outcome remained similar to that of a KAs dosage of more than six tablets per day (Fig. 3).

### Other nutritional and metabolic parameters as secondary outcomes

At study completion, a nutritional parameter assessed by the serum albumin level increased significantly in the LPD–KAs compared with LPD alone group (3.9 ± 0.5 vs. 3.7 ± 0.6 g/dL, *p* = 0.002, respectively). When stratified by diabetes, serum albumin level was also higher in CKD patients with LPD–KAs compared to those with LPD alone in both diabetes (3.9 ± 0.6 vs. 3.7 ± 0.6 g/dL, *p* = 0.002, respectively) and non-diabetes (3.8 ± 0.5 vs. 3.6 ± 0.7 g/dL, *p* = 0.001, respectively). Serum bicarbonate levels also increased significantly in both LPD–KAs (from 22.9 ± 3.4 to 25.2 ± 3.5 mg/dL, *p* < 0.001) and LPD alone groups (from 22.7 ± 4.1 to 23.3 ± 4.6 mg/dL, *p* = 0.006). However, the amount of UACR in patients with LPD–KAs was not different from patients with LPD alone (480.0 [79.1–2024.4] vs. 268.8 [56.6–1255.5] mg/g creatinine, respectively, *p* = 0.06). For CKD-related mineral and bone disorder (MBD) parameters, the serum phosphate levels were comparable between patients with LPD–KAs and LPD alone groups at the end of the study (3.8 ± 1.0 vs. 3.9 ± 1.6 mg/dL, respectively, *p* = 0.11).

## Discussion

We found that LPD–KAs was significantly associated with a lesser decline in kidney function and a lower rate of long-term dialysis initiation than LPD alone among diabetic and non-diabetic CKD patients. A daily dose of KAs equivalent to more than six tablets demonstrated a lower likelihood of rapid CKD progression or commencement of dialysis. However, the spot UACR and serum phosphate levels were similar among CKD patients receiving LPD–KAs and LPD alone.

At present, VLPD supplemented with KAs and LPD alone are the major protein-restricted regimens suggested by the recently launched KDOQI nutrition guideline^9^. However, both strategies are considered for a higher risk of protein-energy wasting unless close nutritional supervision has been maintained. In recognition that the optimal level of dietary protein intake for use in combination with KAs is not well defined^21^, an intermediate regimen of LPD–KAs without intense diet counseling has been proposed. In the present study, we demonstrated that LPD–KAs was significantly associated with a lower rate of eGFR decline and dialysis initiation (HR 0.14; 95% CI 0.10–0.20, *p* < 0.001) when compared with LPD alone among pre-dialysis CKD patients (Table 2). In agreement with our main results but having much fewer participants (139 vs. 1042 patients in our study) and a much shorter duration of follow-up (6 vs. 32.9 months in ours), Picolli et al.^17^ revealed that a simplified approach using LPD–KAs was significantly correlated with a less steep decrease in kidney function among advanced CKD stage 3–5 patients with refractory proteinuria. In similarity to our findings but having only a single arm intervention without a control group in their study, Chang et al.^22^ reported a significantly slower kidney function decline among both diabetic and non-diabetic CKD patients receiving LPD–KAs (− 3.8 mL/min/1.73 m^2^) when compared with LPD alone (− 10.7 mL/min/1.73 m^2^). On the contrary, a previous small study by Mou and colleagues^23^ indicated that there was no significant difference in eGFR after 12 months between LPD–KAs and LPD alone groups. However, all participants enrolled in that study had early stage 1–2 CKD with active glomerulonephritis that is less likely to retard CKD progression from protein restriction alone. Recently, a meta-analysis by Li et al.^24^ also supported our findings that the eGFR change over time was significantly slower among CKD patients receiving LPD–KAs compared with CKD patients obtaining LPD alone with a between-group mean difference of 5.4 mL/min/1.73 m^2^.

There are some indications from the experimental models that dysfunctional amino acid synthesis could intermittently occur in the setting of protein restriction for a period of time despite adequate energy intake. This obscurely insufficient amino acids formation then finally leads to impaired renal gluconeogenesis followed by a worse kidney function prognosis^25,26^. Given that KAs can be re-utilized to form particular functional proteins in the available excess nitrogen during CKD and enter directly into the Kreb’s cycle to produce an endogenous energy fuel^10^, the co-administration of KAs along with LPD may ameliorate these observations and partly provide better kidney protection. Furthermore, supplementation with KAs has been reported to be associated with an enhanced endothelial function through a decrease in human plasma asymmetric dimethylarginine (ADMA) concentrations as well as an alteration in multiple tubular transport mechanisms and thus may be helpful in delaying CKD progression^27,28^.

A supplementation of full-dose KAs containing 0.125 g of keto acids/kg/day or one tablet per 5 kg has been commonly employed in the VLPD approach, whereas the recommended dose to be used along with LPD appears to be undetermined. We found that a dose of more than six tablets of KAs per day, approximated to one tablet per 10 kg, was associated with a lower likelihood of rapid CKD progression or commencement of dialysis (Fig. 3). Although a lower daily dose of KAs (three tablets) than our study has been formerly demonstrated by Khan et al.^29^ for slowing eGFR decline compared with a placebo among the CKD population, the exact amount of daily protein intake in their study was not reported. In consistency with our results but having participants with a more advanced stage of CKD, the dose–response analysis regarding the appropriate dose of KAs supplementation in LPD conducted by Wu et al.^30^ revealed that patients receiving a daily KAs dose of more than 5.5 tablets significantly reduced the need for long-term dialysis. Likewise, an earlier study by Chen and co-workers^31^ in pre-dialysis CKD patients with a serum creatinine of more than 6 mg/dL supported our results that LPD–KAs with the daily dose reduced to one tablet per 10 kg was not inferior to LPD–KAs with a full daily dose corresponding to one tablet per 5 kg with respect to kidney function deterioration over a 6-month period. Based on the opinion of experts from the International Society of Renal Nutrition and Metabolism (ISRNM), a VLPD supplemented with a full dose KAs may not be practical for all nations due to the lack of dietitians experienced in hands-on training, a scarcity of KAs dose–response studies, together with cost barrier-related reimbursement policies among different geographic regions around the world^32,33^. It is likely that LPD–KAs with a reduced dose KAs of one tablet per 10 kg may be alternatively used to enhance the salutary effect of LPD on CKD progression and postpone long-term dialysis in some clinical settings.

In adults with diabetic CKD, a target protein intake of 0.6–0.8 g/kg/day has been suggested without a statement regarding KAs supplementation because there is limited evidence available in relation to their non-diabetic counterparts. In this study, the beneficial effect of LPD–KAs on kidney function was persistent after stratification by diabetes status (Table 3) without any impairment in nutritional status measured by serum albumin levels. A prospective study by Bellizzi et al.^34^ observed that blood urea nitrogen levels and nutritional status, including body weight and serum albumin levels among diabetes after implementing LPD–KAs, significantly improved to the same extent as non-diabetic CKD patients. However, a previous study by Wang and co-workers^35^ was contradictory to ours in that LPD–KAs significantly increased the long-term dialysis risk compared with CKD patients receiving LPD alone in a subgroup of diabetic CKD patients. However, their study recruited only anemic CKD stage 5 patients with multiple comorbidities. Also, the average dose of KAs usage in the study of Wang et al.^35^ was not mentioned, whereas the maximal KAs dose allowed to prescribe was six tablets daily. Overall, we consider that the advantages of LPD–KAs seem to be comparable among both diabetic and non-diabetic CKD, at least in the non-advanced stage of CKD without kidney impairment-related complications. While the data from a previous meta-analysis^36^ revealed that LPD–KAs was associated with a decrease in the serum phosphate level as well as the amount of proteinuria, our study did not demonstrate any significant reductions in either parameter. The extent to which an improvement in CKD-MBD markers or a proteinuria reduction is a consequence of protein restriction or a direct effect of KAs supplementation needs to be confirmed in further well-controlled studies.

Certain strengths and limitations of our study should be taken into account. The present study was relatively large and had a long duration of follow-up with a well-designed study among diabetic and non-diabetic CKD populations. In addition, we used a standard protocol for the monitoring of protein intake as well as a laboratory assessment, including the serum creatinine level. Admittedly, we acknowledge that this study was observational in nature; therefore, the associations do not imply causality. Although most baseline variables that might have potential effect on eGFR decline were not statistically significant, the proportion of diabetes and amount of proteinuria were imbalanced between two groups. Randomized controlled trials are required to ascertain the feasibility and impact of LPD–KAs on kidney function decline as well as other metabolic effects, particularly among the diabetic CKD population. Finally, our study was conducted within a university-based tertiary care center and thus may not be applicable to the whole CKD population, which partly limits its generalizability.

In conclusion, LPD–KAs could retard the progression of CKD and postpone the initiation of long-term dialysis compared with LPD alone in pre-dialysis CKD patients. These beneficial effects were significant among CKD patients receiving the daily KAs dose of more than six tablets. However, there was no improvement in the serum phosphate and albuminuria levels after implementing LPD–KAs.

## Data Availability

The datasets generated and/or analyzed during the current study are not publicly available due to the Personal Data Protection Act, B.E.2562 (2019) but are available from the corresponding author on reasonable request.
